# Whirling in the late Permian: ancestral Gyrinidae show early radiation of beetles before Permian-Triassic mass extinction

**DOI:** 10.1186/s12862-018-1139-8

**Published:** 2018-03-16

**Authors:** Evgeny V. Yan, Rolf G. Beutel, John F. Lawrence

**Affiliations:** 1 Institut für Spezielle Zoologie und Evolutionsbiologie, FSU, 07737 Jena, Germany; 2 Borissiak Paleontological Institute, Russian Academy of Sciences, Profsoyuznaya ul. 123, Moscow, 117997 Russia; 3 Australian National Insect Collection, CSIRO GPO, Box 1700, ACT 2601, Australia and 61 Glenbar Rd., The Palms, QLD, Canberra, 4570 Australia

**Keywords:** Coleoptera, Adephaga, Gyrinidae, New taxon, Permian, Phylogeny, Early evolution

## Abstract

**Background:**

Gyrinidae are a charismatic group of highly specialized beetles, adapted for a unique lifestyle of swimming on the water surface. They prey on drowning insects and other small arthropods caught in the surface film. Studies based on morphological and molecular data suggest that gyrinids were the first branch splitting off in Adephaga, the second largest suborder of beetles. Despite its basal position within this lineage and a very peculiar morphology, earliest Gyrinidae were recorded not earlier than from the Upper Triassic.

**Results:**

*Tunguskagyrus.* with the single species *Tunguskagyrus planus* is described from Late Permian deposits of the Anakit area in Middle Siberia. The genus is assigned to the stemgroup of Gyrinidae, thus shifting back the minimum age of this taxon considerably: *Tunguskagyrus* demonstrates 250 million years of evolutionary stability for a very specialized lifestyle, with a number of key apomorphies characteristic for these epineuston predators and scavengers, but also with some preserved ancestral features not found in extant members of the family. It also implies that major splitting events in this suborder and in crown group Coleoptera had already occurred in the Permian. Gyrinidae and especially aquatic groups of Dytiscoidea flourished in the Mesozoic (for example Coptoclavidae and Dytiscidae) and most survive until the present day, despite the dramatic “Great Dying” – Permian-Triassic mass extinction, which took place shortly (in geological terms) after the time when *Tunguskagyrus* lived.

**Conclusions:**

*Tunguskagyrus* confirms a Permian origin of Adephaga, which was recently suggested by phylogenetic “tip-dating” analysis including both fossil and Recent gyrinids. This also confirms that main splitting events leading to the “modern” lineages of beetles took place before the Permian-Triassic mass extinction. *Tunguskagyrus* shows that Gyrinidae became adapted to swimming on the water surface long before Mesozoic invasions of the aquatic environment took place (Dytiscoidea). The Permian origin of Gyrinidae is consistent with a placement of this highly derived family as the sister group of all remaining adephagan groups, as suggested based on morphological features of larvae and adults and recent analyses of molecular data.

## Background

The Permian-Triassic border 252 mya is an important threshold in the evolution of biota and Coleoptera in particular [1]. This time interval corresponds with the Permian-Triassic mass extinction, “when life nearly died”, and 96% of marine together with 70% of terrestrial vertebrate species became extinct [2]. This event was also the only known example of mass extinction among insects, resulting in 57% of genera and 83% of insect species disappearing from the record [3]. Thus, it has been widely assumed, that representatives of the two most diverse groups of beetles, suborders Adephaga and Polyphaga, comprising 99% of all species, appeared in the Early Triassic at the earliest [4]. For a long time, studies of Permian-Triassic fossil beetles were hindered by insufficient paleontological material and its overall poor preservation, leaving wide space for scientific speculations. Even for a reliable placement of any fossil beetle within these two suborders, a complete body imprint is necessary with at least the ventral aspect preserved in some detail [5].

A Permian origin of adephagan beetles has been suggested [6], but was hitherto insufficiently documented. A basal position of whirligig beetles within Adephaga was proposed, with the Triassic *Triadogyrus* included as an early representative, and possibly also a controversial Permian larva described as *Permosialis* [7]. The discovery of a presumptive Permian species of Gyrinidae underlines a very long evolutionary history of this highly specialized group. It also implies very early splitting events in the suborder Adephaga and in crown group Coleoptera, notably a Permian appearance of Polyphaga. *Tunguskagyrus* gen. Nov*.* (Insecta: Coleoptera) with the single species *T. planus* sp. nov. is described from Late Permian deposits of the Anakit area in Middle Siberia. The genus is assigned to the stemgroup of Gyrinidae, thus shifting back the minimum age of this taxon considerably. *Tunguskagyrus* shows 250 myr of evolutionary stability for a very specialized lifestyle and key apomorphies of epineuston predators and scavengers, which are now characteristic features of Gyrinidae. The current finding clarifies the Paleozoic origin of a “modern” group of beetles, which along with other adephagan and polyphagan taxa flourished in the Mesozoic, and survive until the present day. Apparently the “Great Dying” – the Permian-Triassic mass extinction – had only a limited impact on these groups. These dramatic events took place shortly (in geological terms) after the time when *Tunguskagyrus* lived.

## Results

### Systematic paleontology

INSECTA Linnaeus, 1758.

COLEOPTERA Linnaeus, 1758.

ADEPHAGA Schellenberg, 1806.

GYRINIDAE Latreille, 1810.

*Tunguskagyrus* gen. Nov.

*Tunguskagyrus planus* sp. nov.

#### Etymology

Generic name after Tunguska river and *Gyrinus* Geoffroy, 1762, type genus of the family Gyrinidae. The Latin specific name “planus” refers to the flattened body.

##### Material

Holotype: PIN 5381/32, single specimen, counter print of the complete body impression with distal parts of legs all missing. Repository of Paleontological Institute, Russian Academy of Sciences.

##### Horizon and locality

Upper Permian (Changhsingian), Anakit locality, Lebedevskian Horizon, correlated with Kedrovskian layers of the Maltsevo Formation, Krasnoyarsk region, Russia.

##### Diagnosis

Short, semi-oval head with completely divided compound eyes, dorsal ocular subunits smaller than the lateral ones; short antennae with enlarged, paddle-shaped pedicel and stout proximal flagellomeres; body streamlined and drop-shaped; pronotum very short, transverse and curved; mesoventrite small, as long as mesocoxae, with small anteromedian groove; metacoxae transverse, without coxal plates; six completely exposed abdominal ventrites, the terminal one medially divided.

##### Description

Medium sized, streamlined beetle without pronoto-elytral angle. Elytra very long in relation to head and prothorax. Drop-shaped in dorsal view, appearing flattened. Body length 10 mm, maximum width, 5.1 mm.

Head short, broader than long, rounded anteriorly; region posterior to compound eyes inserted into pronotum. Compound eyes completely divided into larger lateral subunit and smaller dorsal part, the latter in contact with anterior pronotal margin posteriorly; inner margin of lateral ocular subunit reinforced by thick carina; ocelli absent. Head capsule on dorsal side with paired oblique notches, containing antennal insertions. Anterolateral clypeal corners rounded, anterior margin straight; labrum indistinctly visible as very short anteriorly rounded structure. Antenna inserted laterally, anterior to lateral ocular subunit; scape almost spherical; pedicel paddle-shaped, large, distinctly protruding laterally; visible proximal flagellum compact, at least basal 3 flagellomeres short and wide. Mandibles triangular, robust and stout. Lateral lobes of mentum not recognizable, apparently very inconspicuous or absent.

Pronotum strongly transverse, very short, convex posteriorly, concave anteriorly; lateral margins nearly straight, strongly converging anteriorly; moderately sized protruding anterolateral pronotal angles present, apically rounded; pronotal epipleura wide anteriorly, strongly narrowing posteriorly; excavation for prolegs lacking. Propleura exposed, triangular, fairly large, with mesally directed, apically pointed posterior process partly closing procoxal cavities. Prosternum also short and transverse, but distinctly less wide than pronotum; with bead along anterior, posterior and lateral margins; anteriorly slightly concave; posterior edges straight, obliquely converging towards midline; prosternal process very short, almost absent; procoxae transverse, oblique; distal parts of all legs missing (fragment of protibia and metafemur visible, but very incomplete preservation makes them negligible). Elytra wedge-shaped, with rounded apices, finely edged; posteriorly not truncated; surface smooth, without recognizable striae or other sculpture; elytral epipleura anteriorly broad, converging towards elytral apex, lacking excavation for prolegs. Mesoventrite small, with anterior procoxal rests and very small anteromedian groove, lacking transverse suture; posteromedially overlapped by anteromedian process of metaventrite; lateral edges strongly rounded; mesanepisternum triangular, relatively small; mesepimeron fairly wide, very slightly curved, with subparallel margins, slightly widening laterally; mesally forming part of closure of mesocoxal cavities; mesocoxae separated medially, roughly triangular, with anterolateral notch probably fitting with mesotrochantin. Metaventrite large, slightly converging anteriorly, with semicircular excavations for mesocoxae and between them a large triangular anteromedian process; posteromedially with triangular pointed process between mesal metacoxal bases; discrimen not visible; transverse ridge delimiting katepisternum reaches lateral margin of ventrite, mesally converging towards anterior coxal margin. Exposed part of metanepisternum large, triangular, reaching mesocoxal cavity only with narrow mesal apex; metepimeron not recognizable. Metatrochantin not exposed. Metacoxae large, transverse, distinctly reaching beyond posterolateral margin of ventrite; anteriorly not extended, not plate-like; mesally adjacent but mesal walls not fused or connected; plate-like duplicatures, i.e. metacoxal plates absent; distinct sinuate transverse edge likely fitting with anterior metafemoral edge; median coxal lamellae distinct, posteriorly rounded; posterior coxal edge oblique, lateral edge slightly shorter than maximum length close to midline.

Abdomen oval, with six loosely connected ventrites; first completely exposed ventrite corresponds to sternite III, with short median process fitting between mesal coxal lamellae; sternite II only visible laterally as triangular sclerite; sternites III-V almost equally broad, V slightly narrower than III and IV, all three with straight or nearly straight posterior margin. Posterior part of abdomen strongly tapering, with slightly rounded lateral edges and distinctly concave posterior margins; terminal ventrite likely represented by medially divided gonocoxosternum VIII; separation along midline distinct posteriorly but not visible on anterior part. Paired, flat female gonocoxae visible posterior to terminal ventrite; with rounded apical part, reaching elytral apices posteriorly.

## Discussion

### Phylogenetic affinities of *Tunguskagyrus*

**Fig. 1:**
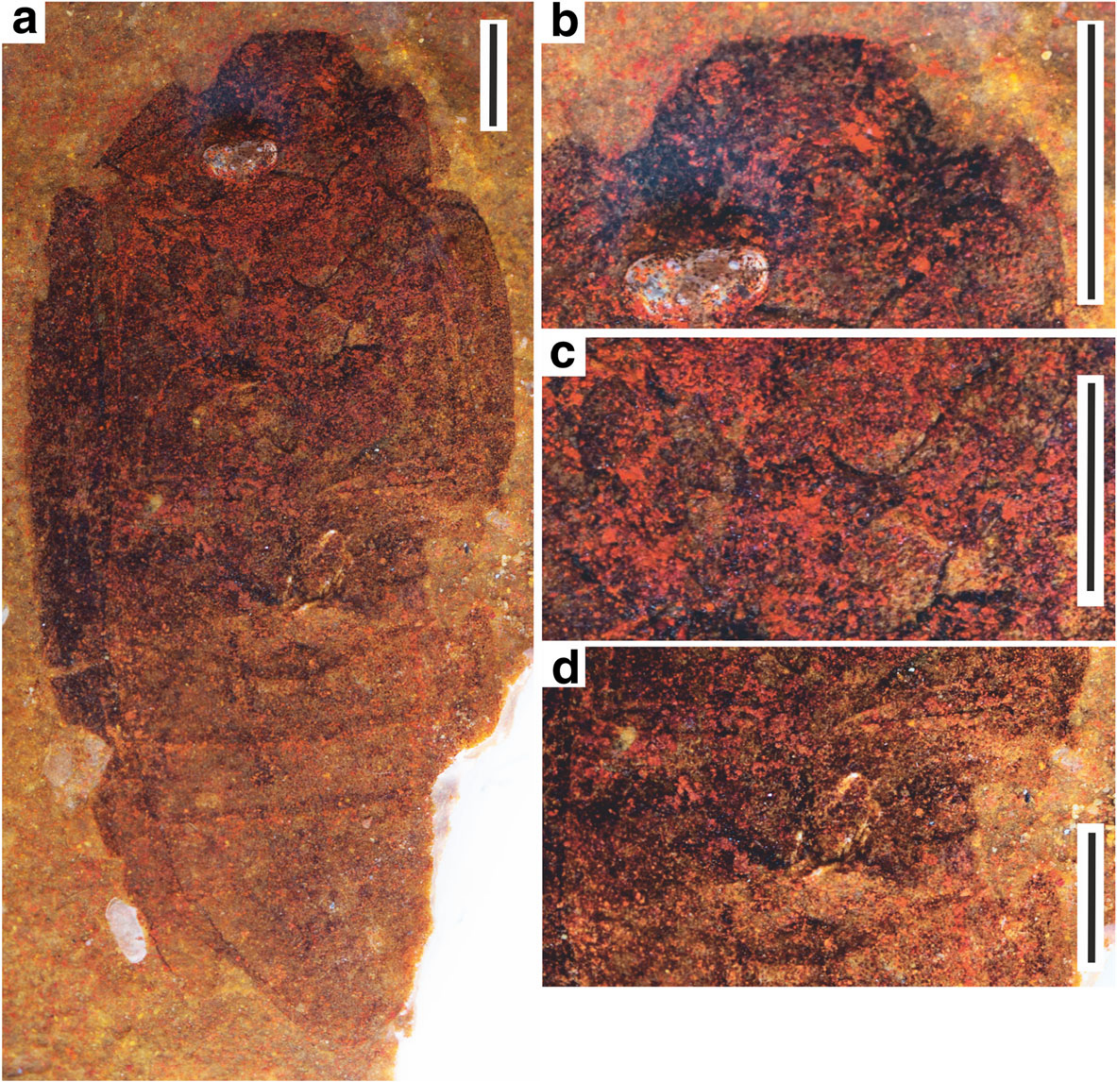
*Tunguskagyrus planus* sp. nov., Habitus. **a** Photograph of the holotype 5381/32. **b** Details of head. **c** Mesoventrite and midcoxae. **d** Metacoxae and the base of abdomen. Scalebars = 1 mm

**Fig. 2:**
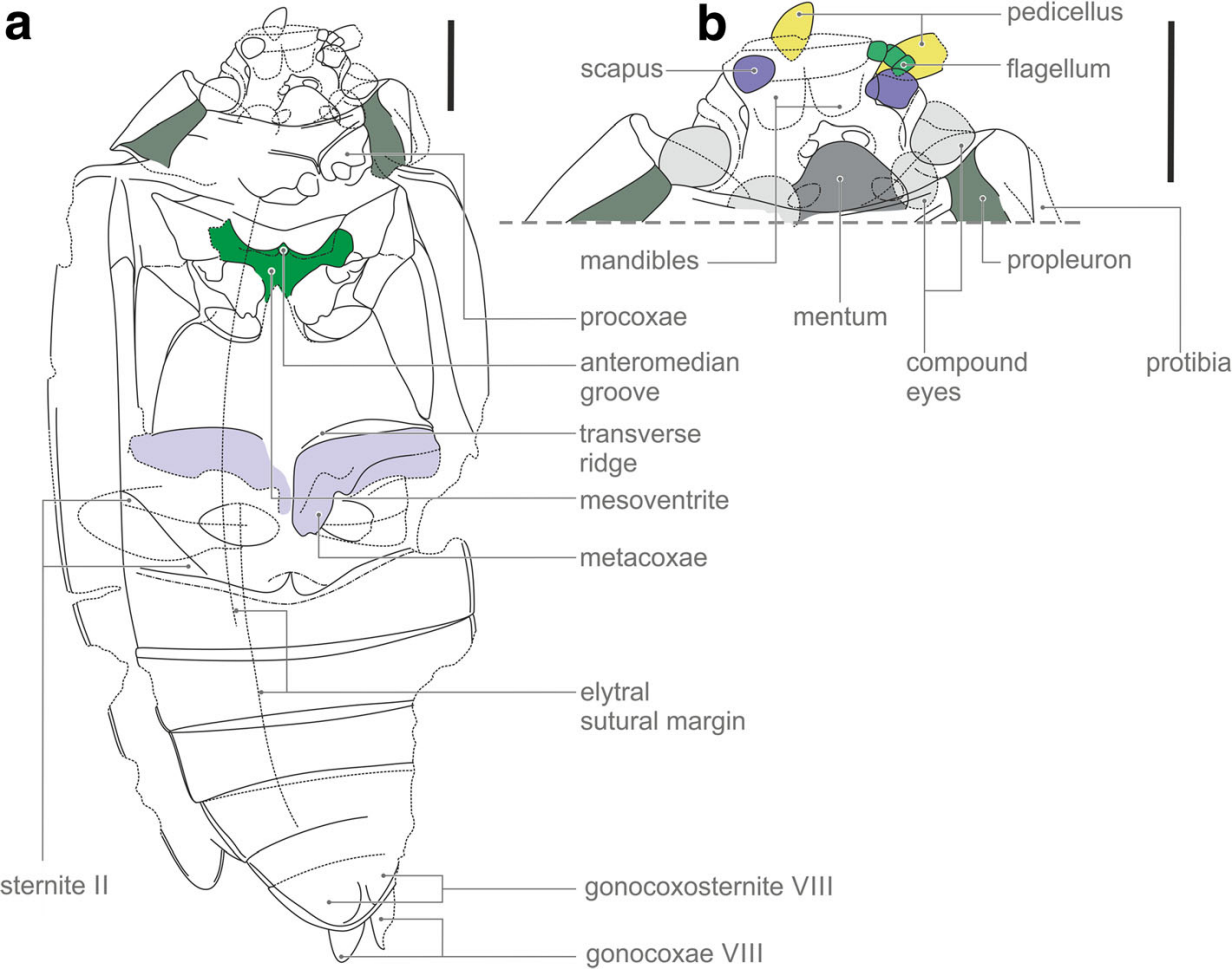
*Tunguskagyrus planus* sp. nov. **a** Interpretative habitual drawing. **b** Interpretation of head structure. Scalebars = 1 mm

*Tunguskagyrus* is a Permian beetle with a striking appearance, differing profoundly from other fossils of the period, notably the taxa assigned to the stem group of Coleoptera by Beutel [4, 5], i.e. Protocoleoptera, Permocupedidae, and Rhombocoleidae. The entire set of features is clearly incompatible with a placement in the “ancestral” Archostemata s.str. [5] or s.l. [8]. This includes the oval body shape, the smooth surface, evenly sclerotized elytra, the lack of a transverse suture of the mesoventrite, the absence of exposed metatrochantins, a metanepisternum not forming a distinct part of the closure of the mesocoxal cavities (only very narrow contact), and the presence of six fully exposed abdominal ventrites (Figs. 1a, 2a). In contrast to *Tunguskagyrus*, the Mesozoic archostematan family Schizophoridae, which was possibly aquatic and had a streamlined body and largely smooth cuticular surface, shows the typical archostematan configuration of the ventral thoracic and abdominal sclerites [5, 8]. This makes a close relationship between the two taxa very unlikely. Moreover, schizophorids do not show any of the specialized cephalic features characterizing the genus described here.

The distinctly exposed propleuron (Figs. 1a, 2a,b) clearly excludes a placement in the megadiverse Polyphaga e.g. [9], according to different studies the sister group of the remaining three suborders e.g. 1, [10]. The comparatively large size, mesocoxae inserted relatively close to each other, and a distinct separation of the meso- and metaventrite are incompatible with a placement in the small suborder Myxophaga, a group characterized by small or very small body size with most species living in hygropetric habitats e.g. [11]. The pterothoracic ventrites are always firmly connected or fused in Myxophaga and also in almost all families of Polyphaga (excl. Scirtoidea and Leiodidae [12]). In contrast, this condition is unknown in Adephaga and also in Archostemata [5, 12].

A large triangular anteromedian process of the metaventrite, as it is found in *Tunguskagyrus*, is a condition typical for beetles of the suborder Adephaga (Fig. 5). This process articulates with the posteromedian part of the mesoventrite [13]. An additional adephagan feature is the large size and transverse shape of the metacoxae, which reach beyond the lateral border of the metaventrite. This feature is secondarily modified in Carabidae, probably constituting autapomorphies of this species-rich terrestrial family [13].

Derived features of the head clearly suggest a placement of *Tunguskagyrus* in the family Gyrinidae (Figs. 1b, 2b). The head in its general shape is similar to what is found in extant and extinct members of this group e.g. [14–16]. A conspicuous shared derived feature of *Tunguskagyrus* and extant Gyrinidae is the complete subdivision of the compound eyes. Another unusual synapomorphy is the modification of the antennal pedicellus as a paddle-shaped structure, presumably set with setae and used for detecting movements of the water surface [17]. An additional antennal apomorphy is the compactness of the flagellum, with stout flagellomeres and apparently hardly reaching beyond the hind margin of the head. Other potential synapomorphies are the short prosternal process, exposed and at least partly separated gonocoxosternites VIII, and exposed flattened gonocoxae VIII see [14, 18, 19] (Figs. 1a, 2a).

Several characteristics of the new genus differ from Mesozoic, Cenozoic and extant Gyrinidae [14]. The most striking difference is the small mesoventrite, in contrast to a flat and extensive sclerite in extant Gyrinidae including the ancestral *Spanglerogyrus* [20]. The mesoventrite of *Tunguskagyrus* also differs from the condition in all other adephagan groups including basal lineages of Carabidae [13]. As in other Gyrinidae, it lacks a large hexagonal groove separated from the anterior edge and usually articulating with a well-developed prosternal process [e.g. [13, 21, 22]. However, in contrast to all extant species of the family a small median groove is present at the anterior margin of the sclerite (Fig. 5). The small size of the mesoventrite of *Tunguskagyrus* implies that this species lacked a large mesothoracic sternotrochanteral muscle M. [51], which creates the main propulsive force of the middle leg in extant Gyrinidae [18, 20]. The presence of a nearly complete transverse ridge of the metaventrite is a symplesiomorphy shared with *Spanglerogyrus* and the non-dytiscoid adephagan groups including Haliplidae [20, 21] and the extinct Triaplidae [23, 24] (Fig. 5). The metacoxae of *Tunguskagyrus* are moderately sized compared to the plate-like extensive ones of extant Gyrinini and Orectochilini [14, 18, 20], without an anterolaterally extended anterior margin. They are more similar to those of *Spanglerogyrus* [20], even though slightly longer at their lateral edge, or also to the metacoxae of Enhydrini e.g. [14] (but compared to these distinctly shorter close to the midline). The moderate size of the metaxocae indicates a smaller size of the coxotrochanteral muscles, which are very large in extant Gyrininae [18, 20, 25] and play an important role in creating the propulsive force of the hindlegs.

The origin of Gyrinidae was estimated to be Late Permian or earlier, the divergence of Spanglerogyrinae from the remaining genera was dated as Triassic, and the divergence time of Heterogyrinae (*Heterogyrus*) from Gyrininae as Late Triassic or Early Jurassic [7]. Considering the available evidence, the most plausible conclusion is to place *Tunguskagyrus* as the sister group of a clade comprising crown group Gyrinidae (including *Spanglerogyrus*) and very likely also *Triadogyrus* and the other Mesozoic members of the family. This monophyletic unit is mainly characterized by an extensive and flat mesoventrite, and correlated with this feature a large mesothoracic sterno-trochanteral muscle is present in extant Gyrininae and Heterogyrinae [20]. The condition of the distal parts of the legs of *Tunguskagyrus* is not documented, but this placement implies that they were not short and paddle-like, as in extant Heterogyrinae and Gyrininae [14, 15, 18, 26], an interpretation also compatible with the comparatively small mesoventrite and metacoxae. The assignment of *Tunguskagyrus* to the stemgroup of Gyrinidae implies that the dorsal eyes were independently reduced in size and shifted dorsad (Figs. 1b, 2b), similar to the condition in Gyrininae [15, 16]. Similarly, the extremely shortened prosternal process in *Tunguskagyrus* and Orectochilini [14] must be a result of parallel evolution. A feature differing from the typical adephagan condition is the lack of lateral lobes of the mentum, which are generally well-developed in extant Adephaga e.g. [22] and even enlarged in Heterogyrinae and Gyrininae e.g. [15, 16]. Nevertheless, a placement in the suborder appears likely, even without considering the obvious structural affinities with extant Gyrinidae.

Gyrinidae, the whirligig beetles, are adapted for a unique lifestyle of swimming in the water surface film [27–29], an unusual habitat sometimes referred to as a “world of the dead and the dying” [29] (Fig. 4). Morphological and recent molecular studies suggest that the family was the first branch splitting off in Adephaga [1, 4, 30, 31], the second largest subgroup of Coleoptera. Despite its basal position within this suborder (sister to all remaining adephagan families) and a very peculiar morphology usually recognizable in the fossil state, until now the earliest extinct Gyrinidae were recorded from Late Triassic deposits of Ukraine [23, 30]. Thus, *Tunguskagyrus* adds more than 60 million years to the minimum age of the family.

### Early evolution of Gyrinidae

Ancestral Permian beetles included in the stem group of Coleoptera (Archostemata s.l.) [8] where very likely associated with wood, with many preserved coleopteran groundplan features, including a tuberculate body surface, broadly closed procoxal cavities, a transverse ridge on the mesoventrite, elytra with a characteristic pattern of window-punctures, an exposed transverse metatrochantin, and five exposed abdominal ventrites, the last one sternite VII, and completely invaginated structures of the following segments, except for a process addressed as ovipositor in the protocoleopteran families Tshecardocoleidae and Moravocoleidae [8, 32].

In striking contrast to this ancestral life style and structural configuration, *Tunguskagyrus* apparently represents a very early invasion of aquatic habitats, with a complex set of adaptations. This includes the streamlined and probably distinctly flattened body, conspicuous modifications of the head, including specifically modified antennae and subdivided compound eyes, and exposed terminal abdominal gonocoxosterna and gonocoxae, the latter probably functioning as steering organs [18]. It is apparent that *Tunguskagyrus* is part of a shifting pattern of late Permian insect communities, with forms much more resembling Mesozoic species than stem group coleopterans (Archostemata s.l.), which were still dominant elements of the beetle diversity in the late Paleozoic e.g. [8, 33].

*Tunguskagyrus* differs distinctly from the aquatic Triaplidae, another extinct adephagan group from the Early Mesozoic (or possibly Late Permian) [23, 30]. This family included medium-sized beetles with an elongated, flattened body, hypognathous head, and large metacoxal plates resembling those of extant Haliplidae [34]. Triaplidae, considered as the first aquatic group of Adephaga [23], showed only moderate adaptations to aquatic habits compared to *Tunguskagyrus*: a stream-lined body and possibly an additional air-storage space under the extensive metacoxal plates e.g. [34]. Apparently lacking adaptations for rapid swimming, triaplids are similar to the extinct archostematan family Schizophoridae in different plesiomorphic features. A lifestyle similar to modern Dryopidae (Coleoptera; Polyphaga) was proposed for members of the small extinct family, with the beetles hanging down from the underside of the water surface film and scraping algae from it [6, 35], similar to feeding habits of the extant polyphagan family Spercheidae (Hydrophiloidea) [36]. The systematic placement of Triaplidae is still unclear [30]. An assignment of *Triaplus sibiricus* to the advanced Dytiscoidea [6] is not supported by convincing apomorphies see e.g. [30].

An entire suite of features of different body parts of “modern” Gyrinidae is related to locomotion in the aquatic environment [18, 37], notably to swimming in the surface film. This includes an enlarged and flat mesoventrite, and a shortened metaventrite resulting from enlarged meso- and metacoxae, which contain large coxo-trochanteral muscles [18]. These features have been observed in the Middle Jurassic *Angarogyrus*, but in clear contrast the plesiomorphic states preserved in *Tunguskagyrus*, bringing this genus closer to the presumptive groundplan of the suborder Adephaga. A mesoventrite of intermediate size is found in the second oldest gyrinid *Triadogyrus sternalis*: the sclerite is definitely larger than that of *Tunguskagyrus* (Fig. 5), but still quite small in comparison to the mesoventrite of later Mesozoic and Cenozoic fossils assigned to Gyrinidae.

The comparatively small metacoxae of *Tunguskagyrus* indicate smaller attachment areas and smaller size of the intrinsic muscle functioning as extensor (M87) after the backward stroke of the hindlegs (M84, M86) [18]. This suggests a limited ability to swim and maneuver fast on or under the water surface in comparison with extant Gyrinidae e.g. [18, 37]. Unmodified mesocoxae also imply that the middle legs were not or only occasionally used for swimming, as it is the case in “modern” Dytiscidae [38]. A noteworthy plesiomorphic feature of the mesoventrite of *Tunguskagyrus* is the presence of an anteromedian pit, which is adapted for receiving the prosternal intercoxal process in other non-adephagan groups of beetles (process strongly reduced in *Tunguskagyrus*). This character is absent in all recent Gyrinidae, but present in almost all fossil representatives of the family, visible as an anteromedial notch (Fig. 5).

Extant Gyrinidae are characterized by a very unusual flight apparatus [18, 22]. Unfortunately, no wings are preserved or other structures allowing conclusions on the flight capacity. However, as flightlessness is apparently a rare exception in the family e.g. [18, 20, 28] and certainly not part of the groundplan, it is likely that *Tunguskagyrus* possessed well developed wings and other elements of the flight apparatus.

## Conclusions

*Tunguskagyrus* confirms a Permian origin of Adephaga, which was hitherto not well supported by published evidence, even though the presence of Trachypachidae and Triaplidae in this period was noted. It is conceivable that a Permian larva described as *Permosialis* also belongs to Gyrinidae and not to Megaloptera. However, this requires a reinvestigation and redescription of the type material [30]. The new genus documents a very early specialization strikingly different from the life habits of stem group Coleoptera e.g. [4, 5, 8, 39], with adaptations to moving on the water surface (Fig. 4) and probably collecting small arthropods caught in the surface film. This may have taken place at about the same time as the switch to an aquatic lifestyle in Triaplidae, which strongly differ in their morphology and very likely also in their life habits. These habitat shifts obviously predated the Mesozoic invasions of the aquatic environment, accomplished by Haliplidae and Dytiscoidea, by members of the non-monophyletic coptoclavids [30], and by hydrophiloids [e.g. *Amphiops* [40]] and other groups of Polyphaga [4, 41, 42]. Compound eyes adapted to living on the water surface is a rare specialization within beetles (see coptoclavids e.g. [30], *Amphiops* [40]). An interpretation as a stepping-stone towards a fully submerged aquatic life was suggested [7, 43] but appears less likely. This would imply secondary fusion of the ocular subunits in subaquatic groups.

*Tunguskagyrus* does not contribute to a clarification of the subordinal relationships in Coleoptera, which are still controversial e.g. [1, 10, 12, 32, 39, 42, 44]. Likewise, it does not provide direct evidence for the interrelationships of the adephagan families. However, the documented Permian origin of Gyrinidae is consistent with a placement of this highly specialized family as the sister group of all the remaining groups of Adephaga, i.e. Haliplidae (possibly closely related with Triaplidae), Dytiscoidea (including a subgroup of Coptoclavidae [30]) and Geadephaga (Trachypachidae and Carabidae). This was suggested based on morphological features of larvae and adults [19, 30], and recent analyses of molecular data also yielded a basal placement of the family [31].

Even though our morphological reconstruction of *Tunguskagyrus* is incomplete due to partial preservation of the type material, it provides a fascinating glimpse of a very early aquatic specialization, very different from the life style of most Permian beetles, underlining a beginning shift in the composition of insect communities before the Permian – Triassic event of mass extinction. Future findings of more complete specimens may confirm (or falsify) the interpretations presented here and further elucidate the life habits of an intriguing Permian beetle species.

## Methods

Specimen of *Tunguskagyrus* was collected by Dr. Sadovnikov G.N. under the programme “Geological and Paleontological studies of Siberia”, in compliance with all sample-collecting procedures, in 1963. Locality description: Anakit (“Anakit” is a group of three closely spaced localities), Lebedevskian Horizon, Krasnoyarsk region, left bank of the river Nizhnyaya Tunguska, right below the mouth of the Anakit river,. Lebedevskian Horizon is correlated with Kedrovskian layers of the Maltsevo Formation in the Tunguska basin [45]. The age of Lebedevskian Horizon was identified as either Permian [46] or Triassic [47]. In later biostratigraphical studies Lebedevskian Horizon is attributed to Changhsingian (252–254 myr) of uppermost Permian [48, 49].

The Anakit locality represents a combination of stagnant and lotic basins during Vyatkian time interval [48], which yields abundant paleontological material including conchostracans [50], bivalves [51], insects [52], amphibians [53] and plants [48]. It seems that intensified of volcanism during the Vyatkian Age did not result in a suppressing effect on the biotas of the basins: only two bivalve species became extinct, while the integral diversity of the assemblages very distinctly increased. The diversity of the Vyatkian leaf flora increased even more in comparison to the preceding Vishkilian Age [48].

**Fig. 3:**
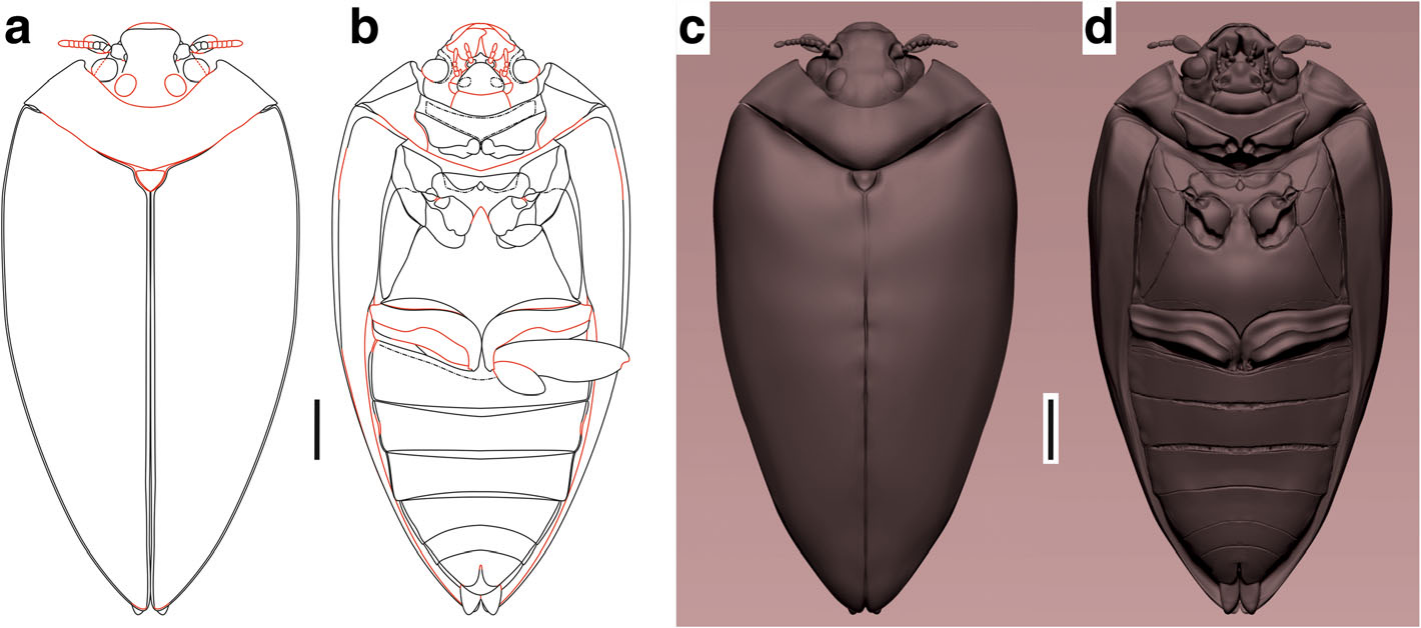
*Tunguskagyrus planus* sp. nov., Habitual reconstructions. **a-b** 2D vector reconstruction. **c-d** 3D reconstruction. Scalebars = 1 mm

**Fig. 4:**
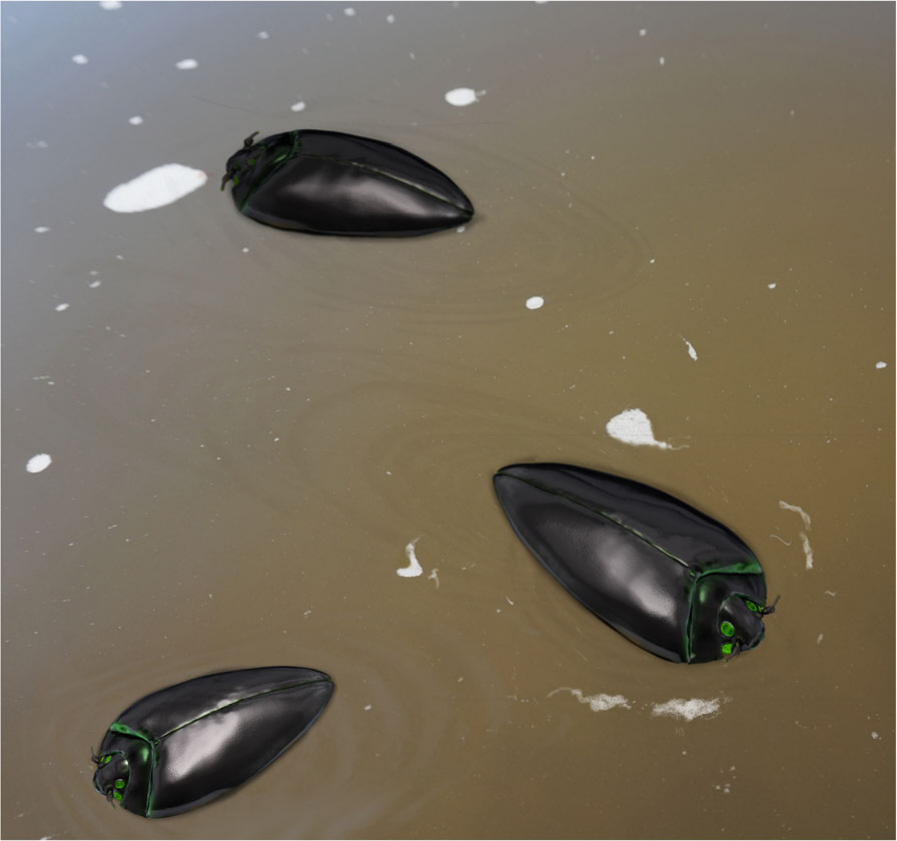
*Tunguskagyrus planus* sp. nov. Artistic reconstruction

**Fig. 5:**
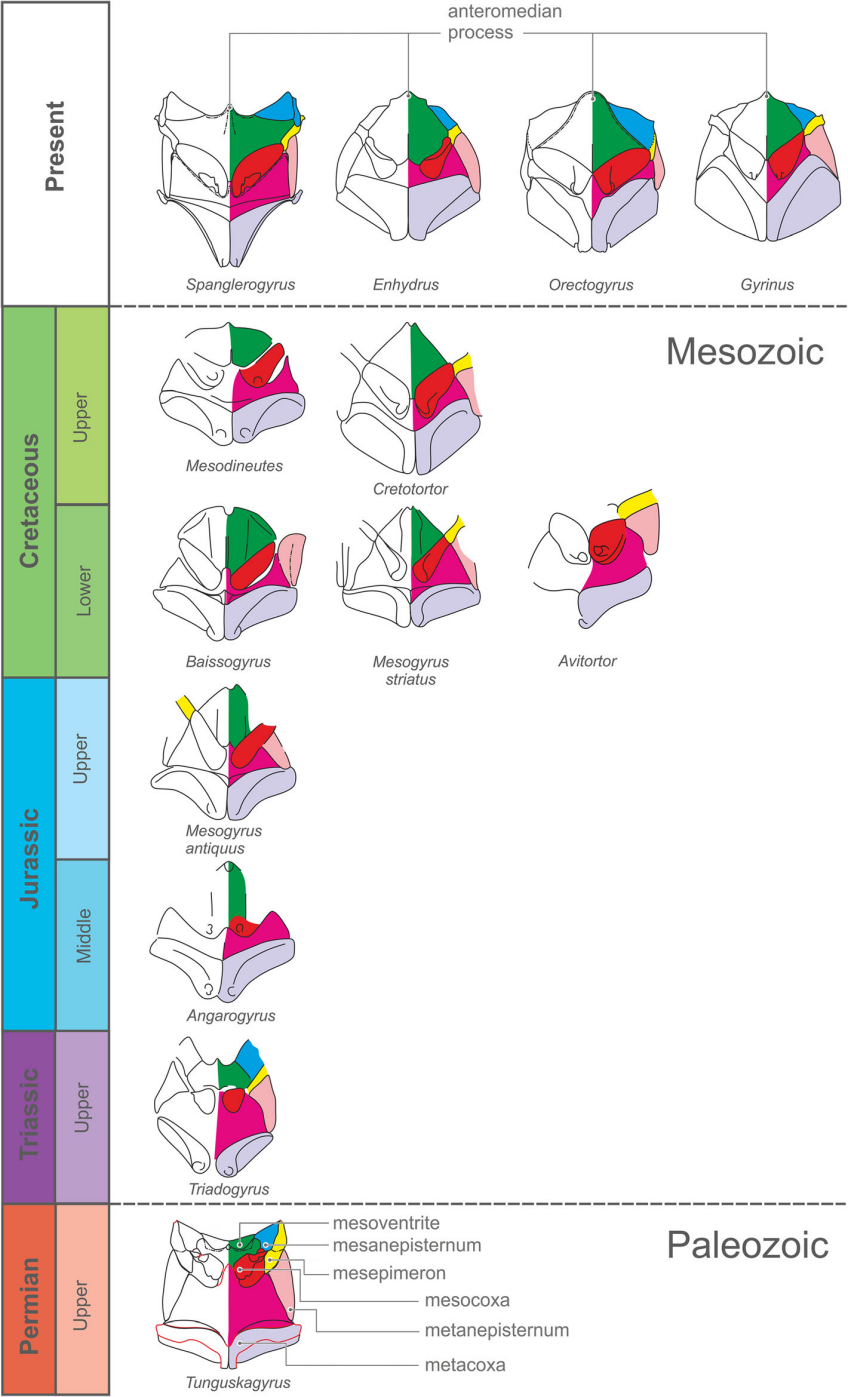
Details of meso- and metathoracic organization in some extinct and Recent Gyrinidae

The specimen of *Tunguskagyrus* was examined dry using microscopes Leica M165C and Zeiss Stemi 2000. The photographs were taken with Leica DFC 425 and Keyence VHX – J20 digital cameras. Line drawings were prepared based on photographs using image-editing software: CorelDRAW X8 and Adobe Photoshop CS. Drawing conventions are as follows: solid line, distinct margin; dashed, indistinct margin and structures overlapping each other; dashed and dotted, fold; dark grey, posteriorly open procoxal cavities. In the 2D reconstructions (Fig. 3a, b and Fig. 4 [*Tunguskagyrus*]) red lines are reconstructed parts, black lines characters restored after type material. Images in Fig. 5 were redrawn after different sources with minor modifications: *Spanglerogyrus* after [20], *Enhydrus* after [14], *Orectogyrus* after [54], *Gyrinus* after [55], *Mesodineutes, Cretotortor, Baissogyrus Mesogyrus striatus* and *M. antiquus* after [56], and *Avitortor*, *Angarogyrus* and *Triadogyrus* after [23]. Gyrinids listed in [7] but not included here are either represented by isolated elytra, or need to be re-examined.

The following measurements were recorded for *Tunguskagyrus* (depending on the state of preservation): total body length; maximum body width; length and width of elytra, head, pronotum, abdomen.

Archostemata (s. str.) is used for the suborder in the sense of Beutel [4] and Beutel and Haas [12], with the majority of extinct Permian families regarded as stem group Coleoptera. Archostemata s.l. refers to the concept of Ponomarenko [8] with Archostemata also including Permian stemgroup beetles (e.g., †Tshecardocoleidae, †Permocupedidae).

## Abbreviations

ACT: Australian capital territory
CSIRO: Commonwealth Scientific and Industrial Research, Canberra, Australia
FSU: Friedrich Schiller University, Institute of Systematic Zoology and Evolutionary Biology, Jena, Thuringia, Germany
GPO: General Post Office
M84: Muscle noto-trochanteralis
M86: Muscle coxa-trochanteralis medialis
M87: Muscle coxa-trochanteralis lateralis
mya: Million years ago
myr: Million years
PIN: Paleontological Institute, Russian Academy of Sciences
QLD: Queensland
RAS: Russian Academy of Sciences
